# Empagliflozin alters lipid metabolism in the myocardium and liver in a prediabetes model with severe dyslipidemia

**DOI:** 10.3389/fphar.2024.1393946

**Published:** 2024-07-04

**Authors:** Denisa Miklankova, Irena Markova, Martina Hüttl, Hana Malinska

**Affiliations:** ^1^ Center for Experimental Medicine, Institute for Clinical and Experimental Medicine, Prague, Czechia; ^2^ First Faculty of Medicine, Charles University, Prague, Czechia

**Keywords:** SGLT2 inhibitors, empagliflozin, cardiovascular disease, lipid metabolism, arachidonic acid, inflammation, ketone body

## Abstract

**Background and aims:**

Recent studies suggest that empagliflozin reduces total and cardiovascular mortality in both diabetic and nondiabetic subjects. Although the exact mechanism is unclear, it is understood to positively affect myocardial energetics, including the metabolism of ketone bodies, lipids, and fatty acids. In this study, we compared empagliflozin effects on lipid metabolism in the heart and liver in a prediabetic rat model with severe dyslipidemia.

**Materials and methods:**

Wistar rats served as the control group, while hereditary hypertriglyceridemic (HHTg) rats were used as a nonobese, prediabetic model. Rats were treated with or without empagliflozin at a dose of 10 mg/kg body weight (BW) for 8 weeks.

**Results:**

In HHTg rats, empagliflozin decreased body weight and adiposity, improved glucose tolerance, and decreased serum triacylglycerols (TAGs) (*p* < 0.001). Empagliflozin decreased the activity and gene expression of the lipogenic enzyme SCD-1 (*p* < 0.001) in the myocardium, which may have led to a decrease in the ectopic accumulation of TAGs and lipotoxic diacylglycerols and lysophosphatidylcholines (*p* < 0.001). Changes in the myocardial phosphatidylcholine/phosphatidylethanolamine ratio (*p* < 0.01) and in the fatty acid profile of myocardial phospholipids may have contributed to the antifibrotic effects of empagliflozin. The anti-inflammatory effects of empagliflozin were evidenced by an increased IL-10/TNFα ratio (*p* < 0.001), a marked decrease in arachidonic acid metabolites (20-HETE, *p* < 0.001), and an increase in PUFA metabolites (14,15-EETs, *p* < 0.001) in the myocardium. However, empagliflozin did not significantly affect either the concentration or utilization of ketone bodies. In the liver, empagliflozin decreased lipogenesis and the accumulation of TAGs and lipotoxic intermediates. Its effect on arachidonic acid metabolites and alterations in n-3 PUFA metabolism was less pronounced than in the myocardium.

**Conclusion:**

Our findings suggest that empagliflozin treatment in the heart and liver reduced the accumulation of neutral lipids and lipotoxic intermediates and altered the metabolism of n-3 PUFA. In the heart, empagliflozin altered arachidonic acid metabolism, which is likely associated with the anti-inflammatory and antifibrotic effects of the drug. We assume that these alterations in lipid metabolism contribute to the cardioprotective effects of empagliflozin in prediabetic states with severe dyslipidemia.

## 1 Introduction

Diabetes is a chronic metabolic disease characterized by the deregulation of glucose and lipid metabolism, a process that aggravates myocardial damage. However, hyperglycemia and glycemic variability are not the only determinants of heightened cardiovascular risk in diabetes. Indeed, research suggests that aggressive glycemic control does not significantly reduce major cardiovascular events (Nusca et al., 2018). Other metabolic disorders such as ectopic lipid accumulation, impaired lipid metabolism, and chronic inflammation are associated with increased cardiovascular risk in diabetic and prediabetic states (Cai et al., 2021).

According to a recent meta-analysis of 10 randomized clinical trials (71,553 participants), novel antidiabetic drugs, including sodium–glucose cotransporter 2 (SGLT2) inhibitors like empagliflozin, reduce total and cardiovascular mortality in diabetic and nondiabetic individuals (Deshpande et al., 2023). Although the exact mechanism behind the cardioprotective effects of SGLT2 inhibitors is not clear, they are understood to positively affect myocardial energetics, including the metabolism of ketone bodies, lipids, and fatty acids. These effects may occur via several independent mechanisms, many of which are not yet fully understood. The beneficial cardioprotective effects of SGLT2 inhibitors are linked not only to their impacts on left ventricular remodeling (Chen and Peng, 2023) and hemodynamics (Gronda et al., 2022), but also to their systemic metabolic effects and direct effects on the heart itself.

Several studies suggest that SGLT2 inhibitors may modulate lipid metabolism independently of their glucose-lowering effects (Szekeres et al., 2021; Yaribeygi et al., 2022). Based on our previous study, which employed a prediabetic model (Hüttl et al., 2021), and other studies performed using models of diet-induced obesity followed by the onset of type 2 diabetes mellitus (Petito-da-Silva et al., 2019; Nasiri-Ansari et al., 2021), empagliflozin treatment decreases hepatic lipid accumulation and improves markers of nonalcoholic fatty liver disease (NAFLD). Clinical studies have also reported improvements in hepatic fat accumulation in diabetic subjects after empagliflozin treatment (Kahl et al., 2020; Chehrehgosha et al., 2021). However, little is known about the effect of SGLT2 inhibitors on lipid accumulation and metabolism in the heart that can be directly associated with the cardiovascular benefits of SGLT2 inhibitors. One *in vitro* study focusing on cardiomyocytes reported that canagliflozin may attenuate myocardial lipotoxicity and inhibit inflammatory signaling pathways in the heart (Zhang et al., 2023).

Recent research suggests that SGLT2 inhibitors may improve cardiac metabolism by shifting myocardial substrate utilization from glucose toward to lipids and ketone body oxidation (Xi et al., 2022). In our previous study, performed using a prediabetic model with serious vascular complications (Hüttl et al., 2020), we confirmed alterations in substrate utilization in the heart after empagliflozin administration. However, the utilization of ketone bodies was not affected.

We hypothesize that lipids, particularly their metabolism in the heart, contribute to the cardioprotective effects of SGLT2 inhibitors, not only by supplying an important source of energy, but also by interfering with various signaling pathways, including pathways involved in the inflammatory response.

In this study, we explore the possible effect of empagliflozin, an SGLT2 inhibitor, on the metabolism of lipids, fatty acids and ketone bodies in the heart, and compare these effects with lipid metabolism in the liver using a prediabetic model, the hereditary hypertriglyceridemic (HHTg) rat (Vrana and Kazdova, 1990; Zicha et al., 2006). This rat strain exhibits genetically determined hypertriglyceridemia, insulin resistance in peripheral tissues, fatty liver, and ectopic lipid accumulation in the absence of obesity, fasting hyperglycemia, or essential hypertension. In our previous study of this rat strain (Miklankova et al., 2021), atherogenic dyslipidemia and myocardial lipotoxicity was associated with impaired glucose oxidation and increased lipogenesis in the heart.

## 2 Methods

### 2.1 Rats and experimental procedure

All experiments were performed in agreement with the Animal Protection Law of the Czech Republic (311/1997), which complies with Council Directive (86/609/ECC) on the use of laboratory animals, and also approved by the Ethics Committee of the Institute for Clinical and Experimental Medicine (protocol number 53/2018). The study was performed using six-month-old male Wistar rats (obtained from Charles River, Germany), serving as the control group, and six-month-old male HHTg rats (provided by the Institute for Clinical and Experimental Medicine, Prague, Czech Republic), serving as the nonobese prediabetic model. Rats were kept in temperature- (22 °C) and humidity-controlled conditions under a 12-h/12-h light/dark cycle with free access to food (maintenance diet for rats and mice; Altromin, Lage, Germany) and drinking water. Wistar and HHTg rats were randomized into groups either treated with empagliflozin or left untreated (Jardiance, Boehinger Ingelheim, Germany). The drug was mixed as part of a standard diet at a dose of 10 mg/kg BW for 8 weeks. At the end of the experiment, rats were decapitated after light anesthesia (zoletil 5 mg/kg BW) in a postprandial state. Aliquots of serum and tissue samples were collected and stored at −80 °C for further analysis.

### 2.2 Analytical methods and biochemical analysis

Serum levels of TAG, glucose, nonesterified fatty acids (NEFAs), as well as total and HDL cholesterol were measured using commercially available kits (Erba Lachema, Czech Republic and Roche Diagnostics, Mannheim, Germany). Serum insulin concentrations were determined using rat ELISA kits (Mercodia AB, Sweden; BioVendor, CZ). Circulating TNFα as well as IL-6, IL-10, hsCRP, and glucagon concentrations were also measured using rat ELISA kits (Bio-Source International, United States; eBioscience, Austria; Alpha Diagnostics International, United States, respectively). Serum and tissue β-hydroxybutyrate concentrations were analyzed using a colorimetric assay kit (Sigma, United States).

For the oral glucose tolerance test (oGTT), blood glucose was determined based on intragastric administration of a glucose load (300 mg/100 g body weight (BW)) after overnight fasting. Blood was drawn from the tail before the glucose load at 0 min and 30, 60, 120, and 180 min thereafter.

In epididymal adipose tissue, basal and adrenaline-stimulated lipolysis was measured *ex vivo* based on the release of NEFA into the incubating medium.

To determine TAGs, diacylglycerols (DAGs), phosphatidylcholine (PC), phosphatidylethanolamine (PE), and lysophosphatidylcholine (LPC) in the myocardium, samples were extracted in dichloromethane/methanol followed by the addition of KH_2_PO_4_; the solution was then centrifuged. The organic phase was evaporated under N_2_; the resulting pellet was then dissolved in an isopropyl alcohol/hexane mixture. Individual lipid classes were separated by thin-layer chromatography (TLC) using hexane/diethyl ether/acetic acid (70:30:1) as a solvent system for neutral lipids (TAG, DAG) or chloroform/ethanol/distilled water/3-ethylamine (30:35:7:35) as a solvent system for polar lipids (LPC, PC, PE). The individual lipid classes were extracted from silica gel and quantified by enzymatic assay (Erba-Lachema, Brno, Czech Republic; Roche Diagnostics, Germany). Concentrations of 14,15-epoxyeicosatrienoic acid (14,15-EET), 20-hydroxyeicosatetraenoic acid (20-HETE), TNFα, and IL-10 in myocardial homogenates were determined using rat ELISA kits (MyBioSource, United States; Invitrogen, Vienna, Austria).

### 2.3 Fatty acid composition and fatty acid desaturase activity

Fatty acid (FA) levels were reported as a percentage of the total fatty acids. FA composition in the heart and liver were determined using TLC. After separation of individual lipid classes and extraction from silica gel (as described above), the samples were converted to fatty acid methyl esters (FAME) using 1% sodium methoxide in dry methanol. FA profiles in separated lipid classes were established by gas chromatography using the Hewlett-Packard GC system with hydrogen as the carrying gas, a flame ionization detector, and a carbowax-fused silica capillary column (Varian, United States). Individual FAME peaks were identified by comparing retention times with those of authentic standards (mix of fatty acids, Restek, United States) (Eder, 1995).

Fatty acid desaturase activity was calculated by determining product/precursor ratios based on the fatty acid composition in individual lipid classes. The following ratios were used to assess the enzyme activities involved in fatty acid metabolism: Δ5-desaturase (20:4n6/20:3n6), Δ6-desaturase (18:3n6/18:2n6), and Δ9-desaturase (16:1n7/16:0). The anti-inflammatory index was calculated as the ratio of selected PUFAs (22:6n3 + 22:5n3 + 20:3n6 + 20:5n3) to 20:4n6 (Strand, et al., 2012).

### 2.4 Relative mRNA expression

Total RNA was isolated from the heart and liver tissue using RNA Blue (Top-Bio, Czech Republic). Reverse transcription and quantitative real-time PCR analysis were performed using the TaqMan RNA-to-C_T_ 1-Step Kit, TaqMan Gene Expression Assay (Applied Biosystems, United States), and the ViiA 7 Real-Time PCR System (Applied Biosystems, United States). Relative expressions were determined after normalization against *Hprt* as the internal reference and calculated using the 2^−ΔΔCt^ method. Results were run in triplicate.

### 2.5 Statistical analysis

Two-way ANOVA was used to analyze the individual and combined effects of treatment and strain for treatment-vs.-strain interactions. All data analyzed were of normal distribution. Fisher’s least significant difference (LSD) *post hoc* test was used for variables showing evidence of treatment-vs.-strain interactions. The test was adjusted for multiple comparisons to determine whether empagliflozin treatment would significantly influence metabolic parameters in HHTg and Wistar strains. The Student’s t-test was used to determine the effect of hypertriglyceridemia on metabolic parameters and lipid metabolism in the heart before empagliflozin treatment. Statistical significance was set at a value of *p* < 0.05. All results are expressed as the mean ± SEM. Statistical analysis was performed using StatSoft Statistica 14 (StatSoft, Czech Republic).

## 3 Results

### 3.1 Characterization of metabolic parameters in HHTg rats

Before empagliflozin administration, there were no differences in initial body weight, glucose, or serum TAGs between the control Wistar and treated Wistar (W + empa) groups or between the prediabetic HHTg and treated HHTg (HHTg + empa) groups. Empagliflozin administration slightly increased food intake (+7%, *p *˂ 0.05) but had no effect on liquid intake. Empagliflozin treatment was confirmed by urinary secretion of glucose in both rat strains.

**FIGURE 1:**
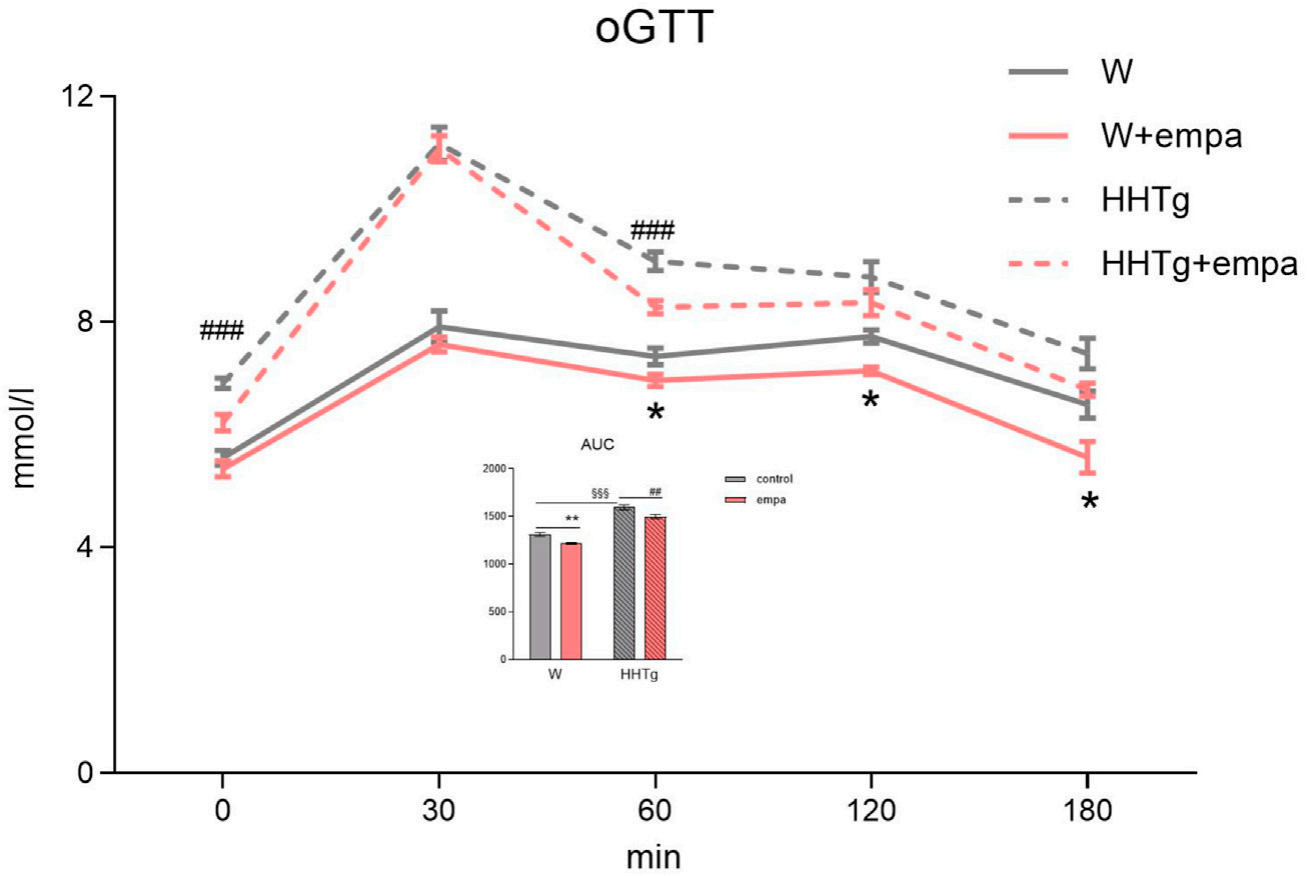
Effects of empagliflozin on glucose tolerance in Wistar control and prediabetic HHTg rats. Data are expressed as the mean ± SEM; *n* = 8 for each group and analyzed by two-way ANOVA with Fisher’s LSD *post hoc* test; * denotes significance reflecting the effect of empagliflozin in Wistar rats; ^#^ denotes significance reflecting the effect of empagliflozin in HHTg rats, ^§^ denotes significance between nontreated Wistar and HHTg rats; * denotes *p* < 0.05, ** denotes *p* ˂ 0.01; ^##^ denotes *p* ˂ 0.01, ^###^ denotes *p* ˂ 0.001; ^§§§^ denotes *p* < 0.001. HHTg, hereditary hypertriglyceridemic; W, Wistar; AUC, area under curve.

**FIGURE 2:**
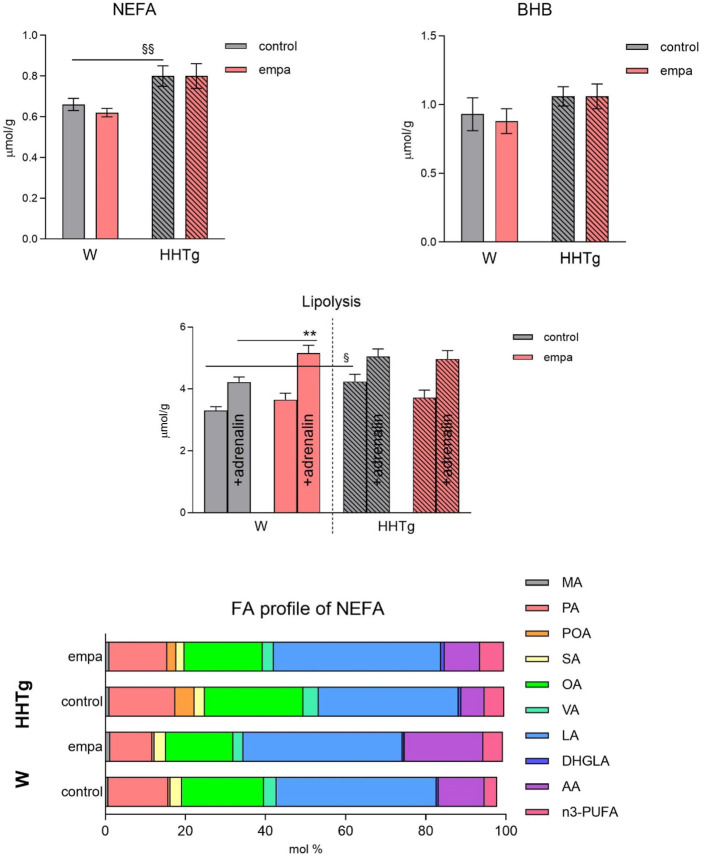
Effects of empagliflozin on serum NEFA, fatty acid profiles of NEFA, lipolysis, and ketone bodies in Wistar control and prediabetic HHTg rats. Data are expressed as the mean ± SEM; *n* = 8 for each group and analyzed by two-way ANOVA with Fisher’s LSD *post hoc* test; * denotes significance reflecting the effect of empagliflozin in Wistar rats; ^§^ denotes significance between nontreated Wistar and HHTg rats; ** denotes *p* ˂ 0.01; ^§^ denotes *p* < 0.05, ^§§^ *p* < 0.01. HHTg, hereditary hypertriglyceridemic; W, Wistar; BHB, β-hydroxy butyrate; MA, myristic acid; PA, palmitic acid; POA, palmitoleic acid; SA, stearic acid; OA, oleic acid; VA, vaccenic acid; LA, linoleic acid; DHGLA, dihomo-γ-linoleic acid; AA, arachidonic acid.

**FIGURE 3:**
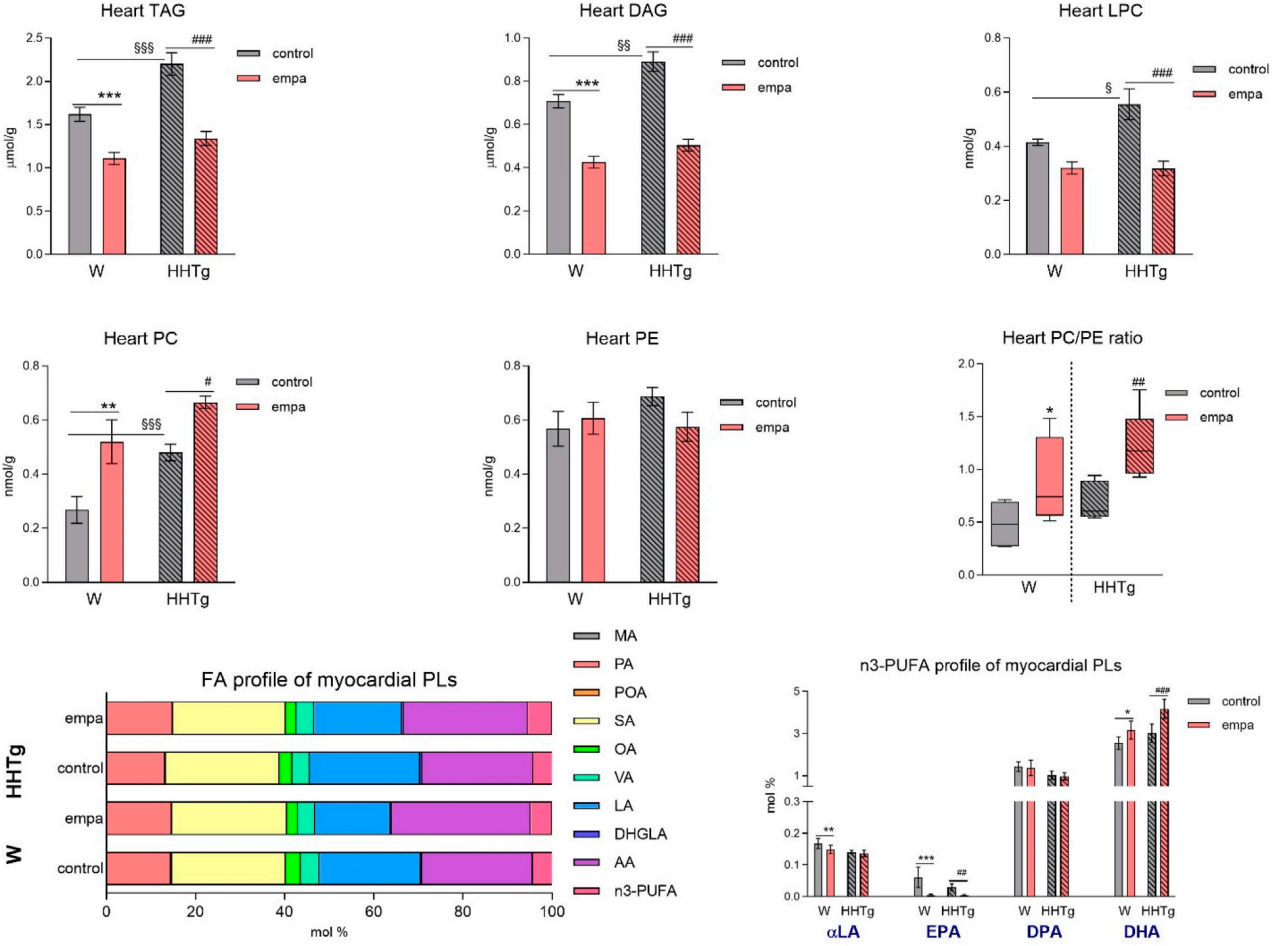
Effects of empagliflozin on the fatty acid profiles of lipids, phospholipids, lipotoxic intermediates, and phospholipids in the myocardium in Wistar control and prediabetic HHTg rats. Data are expressed as the mean ± SEM; *n* = 8 for each group and analyzed by two-way ANOVA with Fisher’s LSD *post hoc* test; * denotes significance reflecting the effect of empagliflozin in Wistar rats; ^#^ denotes significance reflecting the effect of empagliflozin in HHTg rats, ^§^ denotes significance between nontreated Wistar and HHTg rats; * denotes *p* < 0.05, ** denotes *p* ˂ 0.01 *** denotes *p* < 0.001; ^#^ denotes *p* ˂ 0.05,^##^ denotes *p* ˂ 0.01, ^###^ denotes *p* ˂ 0.001; ^§^ denotes *p* < 0.05, ^§§^ denotes *p* < 0.01, ^§§§^ denotes *p* < 0.001. HHTg, hereditary hypertriglyceridemic; W, Wistar; TAG, triacylglycerol; DAG, diacylglycerol; LPC, lysophospholipids; PLs, phospholipids; MA, myristic acid; PA, palmitic acid; POA, palmitoleic acid; SA, stearic acid; OA, oleic acid; VA, vaccenic acid; LA, linoleic acid; DHGLA, dihomo-γ-linoleic acid; AA, arachidonic acid; EPA, eicosapentaenoic acid; DPA, docosapentaenoic acid; DHA, docosahexaenoic acid.

**FIGURE 4:**
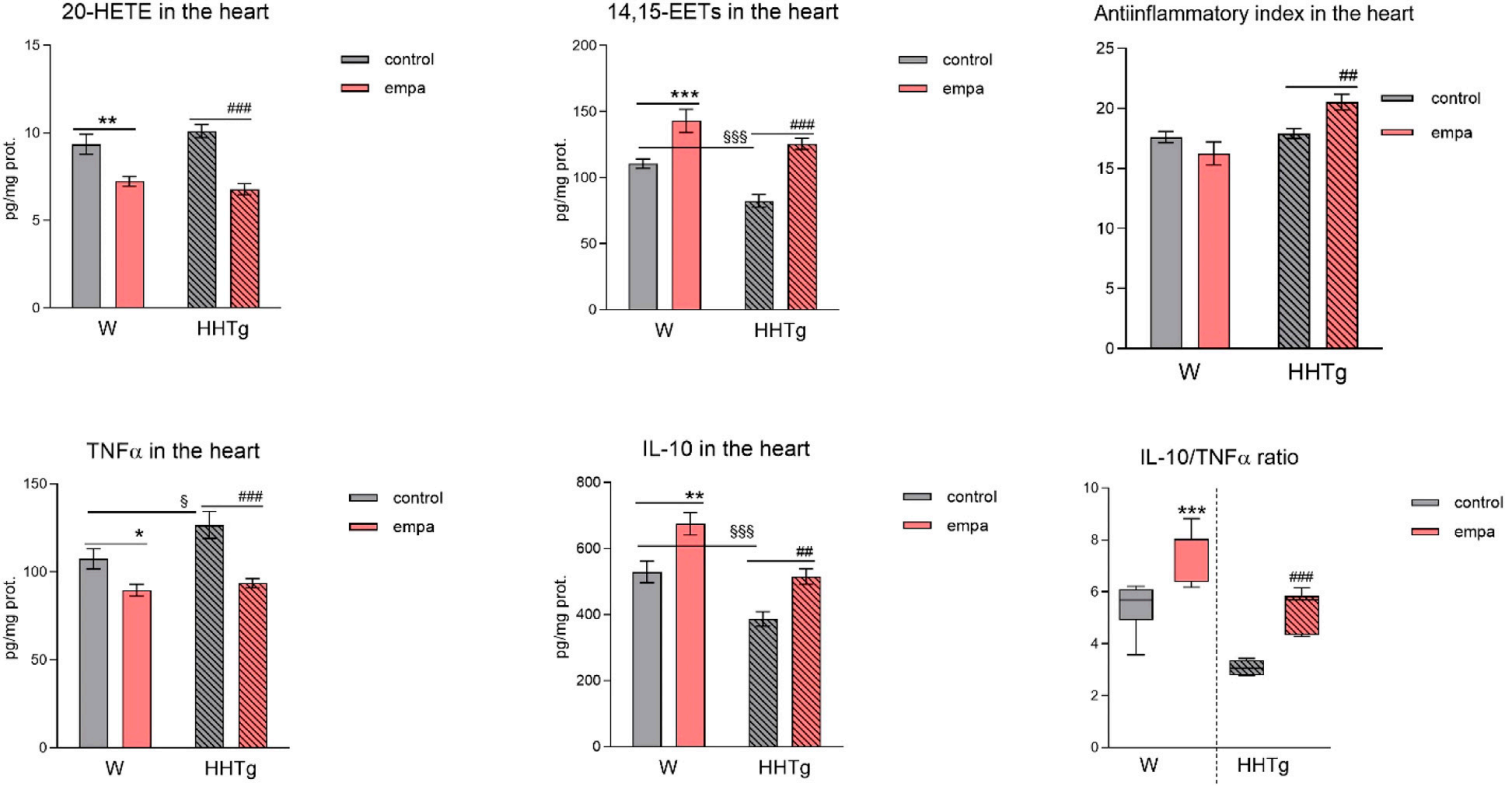
Effects of empagliflozin on inflammatory markers in the myocardium: concentrations of 20-HETE, 14,15-EETs, TNFα, and IL-10 in Wistar control and prediabetic HHTg rats. Data are expressed as the mean ± SEM; *n* = 8 for each group and analyzed by two-way ANOVA with Fisher’s LSD *post hoc* test; * denotes significance reflecting the effect of empagliflozin in Wistar rats; ^#^ denotes significance reflecting the effect of empagliflozin in HHTg rats, ^§^ denotes significance between nontreated Wistar and HHTg rats; * denotes *p* < 0.05, ** denotes *p* ˂ 0.01 *** denotes *p* < 0.001; ^##^ denotes *p* ˂ 0.01, ^###^ denotes *p* ˂ 0.001; ^§^ denotes *p* < 0.05, ^§§§^ denotes *p* < 0.001. HHTg, hereditary hypertriglyceridemic; W, Wistar; HETE, hydroxyeicosatetraenoic acid; EET, epoxyeicosatrienoic acid; TNFα, tumor necrosis factor α; IL-10, interleukin 10.

**FIGURE 5:**
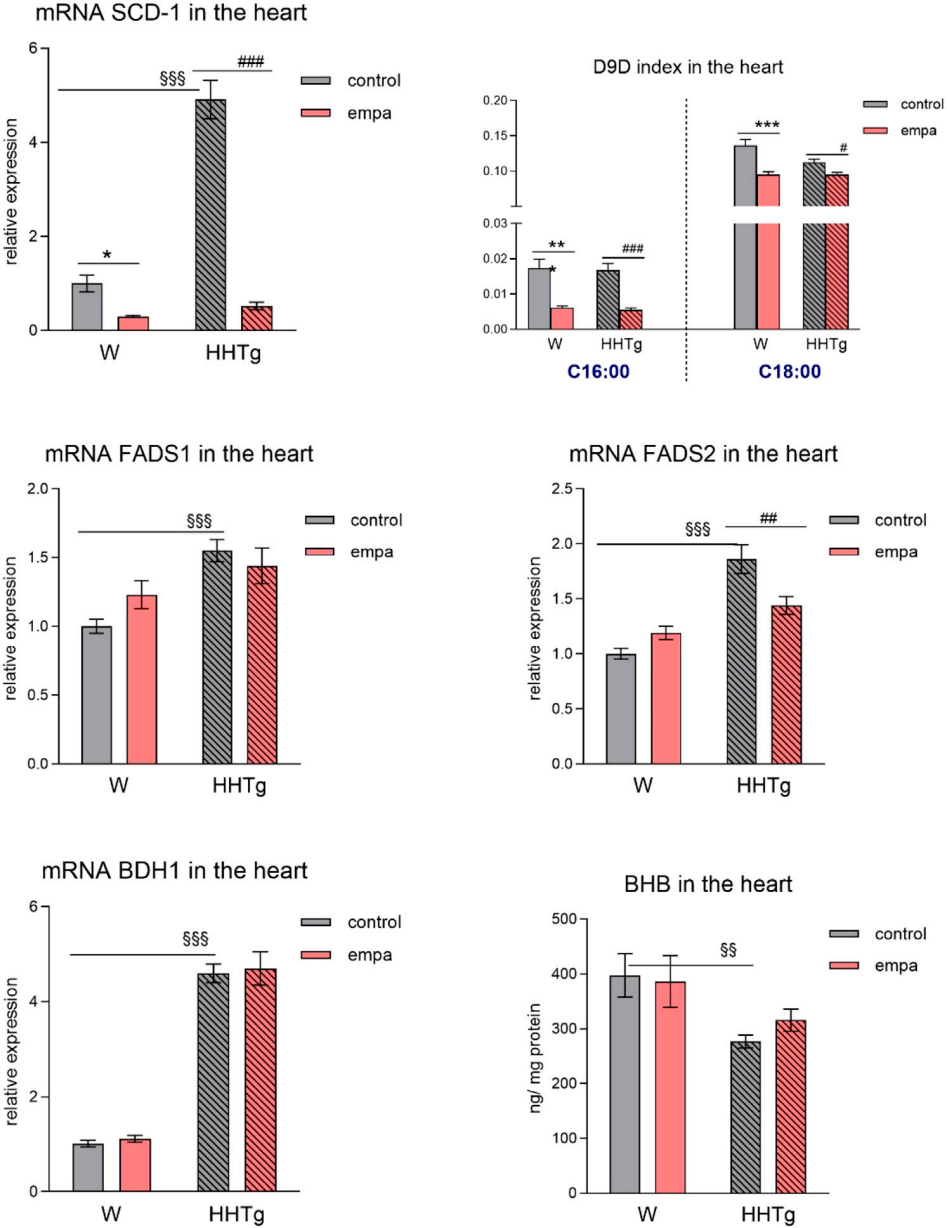
Effects of empagliflozin on parameters of lipid and ketone body metabolism in the myocardium–lipogenesis factors, desaturases, and ketone bodies in Wistar control and prediabetic HHTg rats. Data are expressed as the mean ± SEM; *n* = 8 for each group and analyzed by two-way ANOVA with Fisher’s LSD *post hoc* test; * denotes significance reflecting the effect of empagliflozin in Wistar rats; ^#^ denotes significance reflecting the effect of empagliflozin in HHTg rats, ^§^ denotes significance between nontreated Wistar and HHTg rats; * denotes *p* < 0.05, ** denotes *p* ˂ 0.01, *** denotes *p* < 0.001; ^#^ denotes *p* ˂ 0.05,^##^ denotes *p* ˂ 0.01, ^###^ denotes *p* ˂ 0.001; ^§§^ denotes *p* < 0.01, ^§§§^ denotes *p* < 0.001. HHTg, hereditary hypertriglyceridemic rats; W, Wistar rats; SCD-1, stearoyl-coenzym A desaturase; D9D, delta-9 desaturase; FADS, fatty acid desaturase; BHB, β-hydroxy butyrate; BDH1, β-hydroxybutyrate dehydrogenase 1.

Compared to Wistar controls, untreated HHTg rats exhibited markedly increased serum TAG (*p* < 0.001) and nonfasting glucose (*p* < 0.001) (Table 1), the body weight was significantly decreased in HHTg rats (*p* < 0.001). As expected, hypertriglyceridemia in HHTg rats was associated with impaired glucose tolerance, represented by AUC_0-180_ (*p* < 0.001) during the oGTT test (Figure 1). In addition, untreated HHTg rats exhibited increased NEFA (*p* < 0.05) together with increased basal lipolysis (*p* < 0.01) (Figure 2). We also observed a significant increase in the circulating proinflammatory markers TNFα (*p* < 0.05) and IL-6 (*p* < 0.05) in untreated HHTg rats (Table 2). Further, the accumulation of ectopic myocardial lipids and lipotoxic intermediates was accompanied by an increase in proinflammatory TNFα (*p* < 0.05) and a decrease in anti-inflammatory IL-10 (*p* < 0.01) in the heart in untreated HHTg rats compared to Wistar controls (Figures 3, 4). Increased myocardial TAG accumulation (*p* < 0.01) was associated with a reduction in the FA metabolites 14,15-EETs (*p* < 0.001) and with changes in the FA profiles of myocardial phospholipids. For example, the profile of dihomo-γ-linoleic acid (*p* < 0.001) increased, and the profiles of eicosapentaenoic acid (EPA) (*p* < 0.05) and docosahexaenoic acid (DHA) (*p* < 0.001) decreased in prediabetic HHTg rats (Figures 3, 4). In the heart, relative mRNA expression of the lipogenic enzyme *Scd-1* and the desaturated enzymes *FADS1* and *FADS2* were elevated (*p* < 0.001) (Figure 5) in untreated HHTg rats compared to Wistar controls.

**TABLE 1:** Basal metabolic characteristics.

	W	W + empa	HHTg	HHTg + empa	P_S_	P_T_	P_I_
Body weight (g)	573 ± 8	519 ± 16**	470 ± 9	413 ± 9^**##**^	<0.001	<0.001	n.s.
Visceral adipose tissue weight (mg/100 g)	4.13 ± 0.23	3.08 ± 0.14***	4.28 ± 0.17	3.40 ± 0.11^**###**^	n.s.	<0.001	n.s.
Left ventricle weight (mg/100 g)	0.121 ± 0.002	0.115 ± 0.003	0.127 ± 0.002	0.138 ± 0.013	n.s.	n.s.	n.s.
Nonfasting glucose (mmol/L)	6.78 ± 0.03	6.79 ± 0.12	8.84 ± 0.27	7.90 ± 0.16^**###**^	<0.001	<0.01	<0.01
Insulin (nmol/L)	0.231 ± 0.031	0.168 ± 0.016*	0.311 ± 0.018	0.132 ± 0.013^**###**^	n.s.	<0.001	<0.05
Glucagon (pg/mL)	261.3 ± 36.8	366.7 ± 53.0*	189.4 ± 20.8	215.5 ± 19.3	n.s.	<0.05	n.s.
Serum TAG (mmol/L)	1.28 ± 0.15	0.89 ± 0.12	5.00 ± 0.24	3.43 ± 0.27^**###**^	<0.001	<0.001	<0.01
Serum cholesterol (mmol/L)	2.07 ± 0.11	2.01 ± 0.13	2.07 ± 0.06	1.87 ± 0.06	n.s.	n.s.	n.s.
HDL cholesterol (mmol/L)	0.93 ± 0.06	1.05 ± 0.08	0.92 ± 0.02	0.94 ± 0.02	n.s.	n.s.	n.s.

Two-way ANOVA, results: P_S_, denotes the significance of Wistar vs. HHTg (strain effects), P_T_, denotes the significance of empagliflozin (treatment effects); P_I_, denotes the significance of empagliflozin in both strains (treatment vs. strain interaction). For multiple comparisons Fisher’s LSD, *post hoc* test was used; *, denotes significance reflecting the effect of empagliflozin in Wistar rats; ^**#**^, denotes significance reflecting the effect of empagliflozin in HHTg, rats; *, denotes *p* ˂ 0.05; **, denotes *p* ˂ 0.01; ***, denotes *p* ˂ 0.001; ^**##**^, denotes *p* ˂ 0.01; ^**###**^ denotes *p* ˂ 0.001. Data are expressed as the mean ± SEM; *n* = 8 for each group. HHTg, hereditary hypertriglyceridemic; W, Wistar; TAG, triacylglycerol.

**TABLE 2:** Serum inflammatory parameters.

	W	W + empa	HHTg	HHTg + empa	P_S_	P_T_	P_I_
TNFα (pg/mL)	2.43 ± 0.24	2.18 ± 0.23	3.27 ± 0.21	3.10 ± 0.24	<0.001	n.s.	n.s.
IL-10 (pg/mL)	7.07 ± 0.77	15.18 ± 2.60**	14.62 ± 1.56	20.69 ± 1.87^**#**^	<0.01	<0.01	n.s.
IL-6 (pg/mL)	94.3 ± 4.9	81.4 ± 5.2	116.4 ± 6.2	123.5 ± 7.1	<0.001	n.s.	n.s.
hsCRP (μg/mL)	2.05 ± 0.22	1.45 ± 0.11**	1.53 ± 0.09	1.65 ± 0.15	n.s.	n.s.	<0.05

Two-way ANOVA, results: P_S_, denotes the significance of Wistar vs. HHTg (strain effects), P_T_, denotes the significance of empagliflozin (treatment effects); P_I_, denotes the significance of empagliflozin in both strains (treatment vs. strain interaction). Fisher’s LSD, *post hoc* test was used for multiple comparisons; *, denotes significance reflecting the effect of empagliflozin in Wistar rats; ^**#**^, denotes significance reflecting the effect of empagliflozin in HHTg, rats; ** denotes *p* ˂ 0.01;^#^ denotes *p*˂  0.05. Data are expressed as the mean ± SEM; *n* = 8 for each group. HHTg, hereditary hypertriglyceridemic; W, Wistar; TNFα, tumor necrosis factor α; IL-10, interleukin 10; IL-6, interleukin-6; hsCRP, high-sensitivity C-reactive protein.

### 3.2 Effects of empagliflozin on body parameters, glucose tolerance, and serum lipid parameters

As expected, empagliflozin treatment improved glucose tolerance, which was reflected in significantly decreased serum nonfasting glucose and AUC in the empagliflozin-treated group of rats (Table 1; Figure 1). Markedly reduced serum level of insulin contributed to improvements in glucose tolerance and insulin sensitivity (Table 1). In addition, empagliflozin-treated rats exhibited reduced whole body final weight (−9%), which was associated with a marked reduction in visceral adipose tissue weight (Table 1).

While empagliflozin treatment markedly decreased serum TAG levels in HHTg rats, other serum lipids such as total and HDL cholesterol were not affected (Table 1). Although serum NEFA and ketone body concentrations were not affected by empagliflozin administration, there were significant changes in fatty acid profile in NEFA lipid class (Figure 2). The profile of palmitic (controls *p* < 0.001; HHTg *p* < 0.05), palmitoleic (HHTg *p* < 0.01) and oleic (controls *p* < 0.05; HHTg *p* < 0.001) fatty acid were significantly decreased, while the profile of arachidonic (*p* < 0.001) and n-3 PUFA (*p* < 0.001), in particle EPA (controls *p* < 0.001; HHTg *p* < 0.001) profile were markedly increased in NEFA lipid class after empagliflozin treatment.

### 3.3 Effects of empagliflozin on lipid accumulation and fatty acid profiles in the myocardium

Empagliflozin treatment markedly reduced the accumulation of ectopic neutral TAGs as well as the lipotoxic intermediates DAG and LPC in the myocardium (Figure 3). Myocardial concentrations of individual phospholipids and phosphatidylcholine (PC) were affected in empagliflozin-treated rats. An increase in the myocardial PC/PE ratio after empagliflozin treatment is therefore suitable for use as an anti-inflammatory and antifibrotic parameter.

Our analysis of FA profiles revealed significant changes in myocardial phospholipids after empagliflozin treatment. The decreased profiles of palmitoleic (*p* < 0.001) and dihomo-γ-linoleic (controls *p* < 0.05; HHTg *p* < 0.001) fatty acids may have contributed to improvements in insulin sensitivity in the heart, while the markedly increased profiles of n-3 PUFA (controls *p* < 0.05; HHTg *p* < 0.001) and arachidonic (*p* < 0.001) fatty acid are likely more related with FA metabolism and signaling pathways in the heart. We observed a decrease in the profile of EPA (controls *p* < 0.001; HHTg *p* < 0.01). However, the profile of DHA (controls *p* < 0.05; HHTg *p* < 0.001) in myocardial phospholipids significantly increased in empagliflozin-treated rats (Figure 3).

### 3.4 Effects of empagliflozin on inflammatory markers

Arachidonic acid metabolites, such as proinflammatory 20-HETE, decreased, while anti-inflammatory 14,15-EET increased in the heart after empagliflozin administration (Figure 4). In addition, in empagliflozin-treated HHTg rats, the anti-inflammatory index calculated based on selected fatty acids significantly increased. The anti-inflammatory effect of empagliflozin in the heart may also be due to reduced myocardial levels of TNFα and increased myocardial levels of IL-10 in rats treated with empagliflozin (Figure 4). Anti-inflammatory effects of empagliflozin in the myocardium was accompanied by changes in serum inflammatory markers. For instance, the serum level of IL-10 significantly increased in both empagliflozin-treated groups of rats (Table 2).

### 3.5 Effects of empagliflozin on lipid and ketone body metabolism in the myocardium

Further, in the myocardium, empagliflozin administration significantly decreased relative mRNA expression of *Scd-1*, an important lipogenic enzyme, and decreased the D9D index, which reflects Scd-1 activity in both rat strains (Figure 5), highlighting the effect of empagliflozin on lipogenesis. Empagliflozin administration did not affect the relative gene expression of the desaturated enzyme *FADS1*, whereas the relative gene expression of *FADS2* significantly decreased in empagliflozin-treated HHTg rats (Figure 5). Compared to controls, HHTg rats exhibited decreased ketone body concentration in the heart, which was associated with significantly elevated relative gene expression of the *BDH1* enzyme. However, empagliflozin treatment had no significant effect on either myocardial β-hydroxy butyrate (BHB) concentration or mRNA gene expression of β-hydroxybutyrate dehydrogenase 1 (*BDH1*) (Figure 5).

### 3.6 Effects of empagliflozin on the metabolism of hepatic lipids, lipids, and fatty acids

**FIGURE 6:**
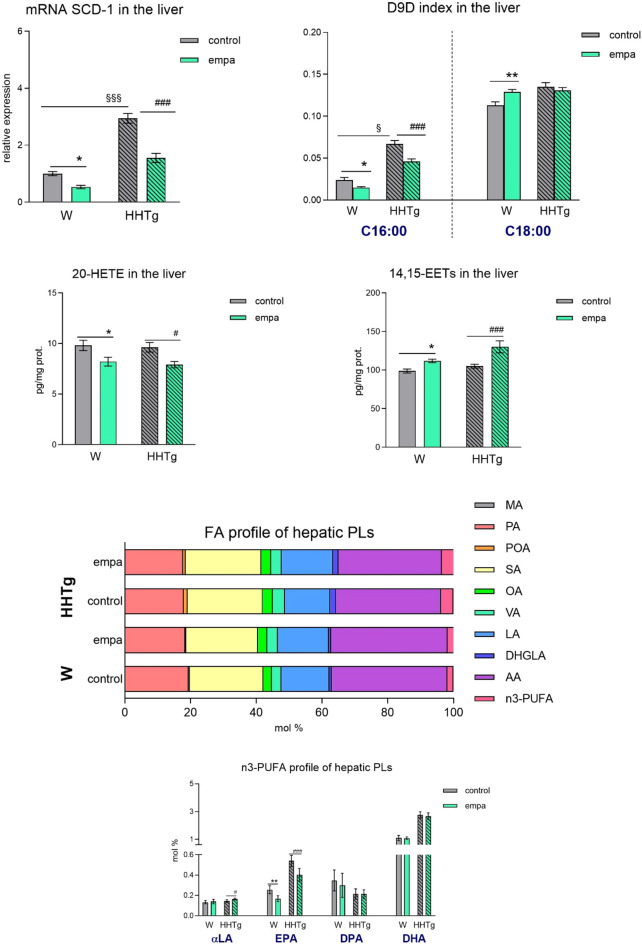
Effects of empagliflozin on lipid and inflammatory markers in the liver: relative expression and activity of Scd-1, 20-HETE, 14–15 EET, and fatty acid profiles of hepatic phospholipids in Wistar control and prediabetic HHTg rats. Data are expressed as the mean ± SEM; *n* = 8 for each group and analyzed by two-way ANOVA with Fisher’s LSD *post hoc* test; * denotes significance reflecting the effect of empagliflozin in Wistar rats; ^#^ denotes significance reflecting the effect of empagliflozin in HHTg rats, ^§^ denotes significance between nontreated Wistar and HHTg rats; * denotes *p* < 0.05, ** denotes *p* ˂ 0.01; ^#^ denotes *p* < 0.05, ^###^ denotes *p* ˂ 0.001; ^§^ denotes *p* < 0.05, ^§§§^ denotes *p* < 0.001. HHTg, hereditary hypertriglyceridemic rats; W, Wistar rats; SCD-1, stearoyl-CoA desaturase 1; D9D, delta-9 desaturase; HETE, hydroxyeicosatetraenoic acid; EET, epoxyeicosatrienoic acid; PLs, phospholipids; MA, myristic acid; PA, palmitic acid; POA, palmitoleic acid; SA, stearic acid; OA, oleic acid; VA, vaccenic acid; LA, linoleic acid; DHGLA, dihomo-γ-linoleic acid; AA, arachidonic acid; EPA, eicosapentaenoic acid; DPA, docosapentaenoic acid; DHA, docosahexaenoic acid.

In the liver, reduced hepatic lipogenesis after empagliflozin administration may have contributed to a reduction in the lipogenic enzyme Scd-1 and relative mRNA expression and activity calculated as the D9D index (Figure 6). The proinflammatory metabolite 20-HETE slightly decreased in the liver in both empagliflozin-treated groups of rats, while the anti-inflammatory metabolite 14,15-EET increased slightly in controls, and markedly so in the prediabetic group of rats. Empagliflozin administration in the liver altered the FA profiles of hepatic phospholipids, decreased the profile of palmitoleic acid (controls *p* < 0.05; HHTg *p* < 0.001), and increased the profile of linoleic acid (controls *p* < 0.05; HHTg *p* < 0.001). Although the n-3 PUFA profile was not affected, detailed analysis of the profile in individual n-3 PUFA revealed a decreased profile of EPA and increase in the profile of α-linolenic fatty acid (Figure 6). However, the changes in FA composition in hepatic phospholipids after empagliflozin treatment were less pronounced compared to the changes we observed in the myocardium.

## 4 Discussion

It is becoming increasingly evident that the cardiovascular benefits observed after treatment with SGLT2 inhibitors are not exclusively related to glycemic control and reduced glucotoxicity. Although the mechanism underlying these benefits is poorly understood, a mounting body of research suggests that modulations in inflammation and energy metabolism play a key role.

A reduction in glucose calories induced by SGLT2 inhibitors such as empagliflozin is consistent with increased lipid and ketone body mobilization (Szekeres et al., 2021). In our study, empagliflozin treatment improved serum dyslipidemia, first of all decreased serum triglycerides levels, but had no effect on circulating NEFAs or serum ketone bodies. HHTg rats typically exhibit chronically elevated levels of circulating NEFAs. However, in our study, treatment with empagliflozin did not further affect these NEFA values. Despite no change in NEFA concentration, empagliflozin significantly altered the FA profile of the NEFA lipid class, which is likely to have had a major impact on the transport and uptake of fatty acids in the myocardium. Qualitative changes in the FA profiles of NEFAs are believed to contribute to the effect of empagliflozin on the metabolism of adipose tissue. Although empagliflozin only slightly affected lipolysis in our study, it may have altered the composition of FAs released from adipose tissue. Cardiac energy metabolism depends on the availability, concentration, and FA profiles of circulating NEFAs. As previously described (Markova, et al., 2019), our prediabetic rat strain exhibits chronically elevated NEFA levels accompanied by qualitative changes in the NEFA lipid class, which may be partially attributed to a reduction in the n-3 PUFA profile. In humans, according to meta-analysis with type 2 diabetes patients SGLT2 inhibitors as monotherapy given only minor effect on serum lipid profile (Lazarte et al., 2021). In type 2 diabetes patients, significant increases in total, LDL and HDL cholesterol were observed in SGLT2 inhibitors treated-patients. Moreover, a reduction of serum triglycerides is described in both clinical and preclinical studies with SGLT2 inhibitors (Salvatore et al., 2022). The increase in HDL cholesterol and decreased in triglycerides is mediated partly through improved insulin secretion and sensitivity.

Ectopic lipid deposition and myocardial lipotoxicity play important roles in the development of myocardial damage, both structurally and functionally, and have been shown to affect substrate utilization and the development of cardiomyopathies (Xi et al., 2022). Although HHTg rats do not exhibit left ventricular hypertrophy, they do tend to accumulate lipid and lipotoxic intermediates in the heart, which is associated with accelerated lipogenesis. A previous study of ours demonstrated that this rat strain is characterized by an imbalance in substrate utilization in the myocardium, reflected in reduced glucose oxidation and increased FA oxidation (Miklankova et al., 2021). This observation tallies with a human study of diabetic patients, which found that increased intracardiac TAG concentration is associated with left ventricular remodeling and reduced cardiac energy (Levelt, et al., 2016).

In our study, empagliflozin treatment led to a reduction in the accumulation of myocardial lipids, which is a predictor of improved myocardial energy metabolism. This reduction was also observed in neutral TAGs as well in lipotoxic DAGs and LPCs. These lipotoxic metabolites interfere with intracellular signaling pathways, resulting in the impairment of cell function, partial impairment of insulin signaling, and enhanced inflammation (Ormazabal, et al., 2018). It has even been suggested that DAGs are an independent predictor of cardiac fibrosis (Marin-Royo, et al., 2018). Thus, a reduction in lipotoxic intermediates likely contributes to the anti-inflammatory effect of empagliflozin in the heart and consequent improvements in myocardial insulin resistance.

Our results indicate that a reduction in the lipogenic enzyme Scd-1 along with changes in the FA profile of NEFAs may contribute to decreased accumulation of myocardial lipids. Scd-1 is an important factor in maintaining efficient cardiac lipid metabolism through the lipogenesis of cardiomyocytes and can also improve cardiac function by shifting substrate utilization in the myocardium (Balatskyi and Dobrzyn, 2023). Therefore, decreased gene expression of *Scd-1* after empagliflozin treatment not only contributes to a reduction in the accumulation of myocardial lipids, but can also optimize the utilization of glucose and lipids in the myocardium. Based on our previous study and others (Ormazabal et al., 2018; Miklankova et al., 2021), metabolic flexibility in the myocardium is impaired in an insulin-resistant state, which is associated with an imbalance between lipid and glucose uptake by cardiomyocytes, leading to intramyocardial accumulation. SGLT2 inhibitors reduce lipogenesis via several cellular pathways, including Scd-1 and SREBP-1c. They are also understood to stimulate FA oxidation. However, the effect of empagliflozin on gene expression involved in FA oxidation in the myocardium is unclear.

Composition of the phospholipids PC and PE may contribute to a reduction in lipid accumulation in the myocardium after empagliflozin treatment. The PC/PE ratio influences mitochondrial energy metabolism (van der Veen, et al., 2017) and can modulate the organization of lipid rafts in membrane regions (Gholam, et al., 2023). By altering the PC/PE ratio, empagliflozin can modulate membrane fluidity and inflammatory responses while mediating their antifibrotic effects. Precisely how SGLT2 inhibitors affect phospholipids in the myocardium is not known. However, the authors of one study of diabetic mice found that dapagliflozin altered the density of lipid rafts in the membranes of proximal tubular cells (Gholam et al., 2023).

According to our results, the effect of empagliflozin treatment on the myocardium is likely associated with the metabolism of n-3 PUFA and arachidonic acid and the generation of bioactive metabolites originating from these metabolic lipid pathways. We observed that empagliflozin influenced inflammatory metabolites generated by lipid metabolism, which is associated with altered arachidonic acid and n-3 PUFA metabolism. Interestingly, the EPA profiles of myocardial phospholipids decreased, which could be related to the generation of anti-inflammatory EET metabolites. However, the EPA profile in circulating NEFA increased. Our results support the premise that EPA is more likely to be transported into the myocardium, where it is then metabolized into anti-inflammatory metabolites. For FAs to be metabolized, they must be released from the membrane. This means that an increase in the profile of membrane-bound arachidonic acid might not necessarily lead to the formation of proinflammatory metabolites. Our results are in line with previous studies of diabetic rats, where arachidonic acid metabolites were shown to worsen cardiac function (Yousif, et al., 2009) and contribute to the development and progression of cardiac hypertrophy (Zordoky and El-Kadi, 2008). In our study, an increase in the arachidonic acid profile and a decrease in the EPA profile in myocardial phospholipids resulted in a decrease in HETEs and an increase in EET concentrations. The two eicosanoids 20-HETE and 14,15-EET play an important role in the pathogenesis of myocardial damage, modulating inflammation and oxidative stress during the development of diabetic complications and in the heart. In addition, EETs help to regulate the cardiovascular system by exerting a vasodilatory and natriuretic effect. For instance, 20-HETE can affect insulin signaling. The potent effect of 20-HETE on the cardiovascular system involves stimulating the contractility and proliferation of smooth muscle cells, as well as activating endothelial dysfunction and inflammation. Dietary DHA can reduce the plasma level of 20-HETE, highlighting the interaction between DHA metabolites and HETEs (Xi et al., 2022). The mechanism by which empagliflozin affects 20-HETE in the myocardium is not yet understood. However, multiomics analysis of a rat model with diabetic cardiomyopathy indicates that the perturbation of PUFA biosynthesis and arachidonic acid metabolism are responsible for the effects of empagliflozin (Xi et al., 2022). In our study, the decreased profile of oleic acid in myocardial phospholipids is consistent with the theory that it serves as a preferential substrate in the regulation of FA oxidation. Although the mechanism is poorly understood, it may be potentially linked to PPARα activation (Finck, et al., 2003).

In addition to affecting inflammatory metabolites generated by lipids, empagliflozin treatment also has a positive effect on the myocardial levels of the inflammatory cytokines TNFα and IL-10. In our study, the decrease in myocardial TNFα was not associated with a decrease in myocardial *TNF*α gene expression or with alterations in the circulating levels of TNFα. On the other hand, the increase in anti-inflammatory IL-10 in the myocardium was also evident in serum. According to one study, a significant interrelationship exists between IL-10, TNFα, and heart failure (Kaur, et al., 2009). IL-10 suppresses the production of TNFα and other proinflammatory cytokines such as IL-1, IL-6, and IL-8. However, TNFα is a mediator of cardiac pathology via inflammation pathways and the activation of apoptosis. Cardiac myocytes isolated from rat and mouse hearts were shown to contain TNFα, which suggests that heart cells are capable of producing TNFα (Kaur et al., 2009). Thus, the effect of empagliflozin may be directly attributed either to TNFα production in the heart or to the release of TNFα from adipose tissue, in particle epicardial tissue. Consistent with our results, some studies have drawn attention to the possible anti-inflammatory properties of SGLT2 inhibitors. A reduction of gene expression in proinflammatory cytokines, including *IL-6* and *TNF*α, after empagliflozin treatment was demonstrated in the hearts of obese diabetic rats (Aragon-Herrera, et al., 2019). In studies involving type 2 diabetic patients, treatment with canagliflozin has lowered the serum levels of leptin and IL-6 (Garvey, et al., 2018), while an increase in the serum level of anti-inflammatory IL-10 has been observed after empagliflozin treatment (Iannantuoni, et al., 2019). The modulation of inflammatory signaling pathways involving NFκB inhibition and activation of AMPK signalization are understood to contribute to the anti-inflammatory properties of empagliflozin (Hawley, et al., 2016). In addition, several studies have shown that SGLT2 inhibitors can affect macrophage polarization from the M1 to the M2 subtype (Koyani, et al., 2020), albeit not in the context of cardiovascular and myocardial injury. In agreement with our results, changes in the FA profiles of myocardial phospholipids, particularly an increase in the n-3 PUFA profile, has been linked to the influence of macrophage polarization. Thus, it is possible that reduced TNFα in the heart after empagliflozin treatment is associated with decreased M1 macrophages. According to this theory, the effect of macrophage membrane polarization is to reduce infiltration of inflammatory macrophages in the heart. In summary, the anti-inflammatory effect of empagliflozin is probably either reflected in indirect improvements in metabolism or direct modulation of inflammatory signaling pathways in the heart along with inflammatory cytokine release.

Empagliflozin may also have a cardioprotective effect on ketone bodies in the myocardium (Packer, 2023). In our study, empagliflozin treatment had no significant effect on either BHB concentration or utilization in the myocardium. However, empagliflozin administration did lead to a partial normalization of the reduction in myocardial BHB levels. That empagliflozin did not affect circulating concentrations of BHB, which are determinants of myocardial concentrations, may partly explain this effect. However, our results are also consistent with a general trend observed in human studies, which is that circulating ketone bodies do not increase after the administration of SGLT2 inhibitors in nondiabetics (Marilly, et al., 2022). Ketone bodies are believed to have antioxidant, anti-inflammatory, and antifibrotic effects on the heart and may also function as important signaling molecules. These effects are probably used more than as an alternative energy sources. In our previous study, which employed a prediabetic model with serious vascular complications (Huttl et al., 2020), we noted alterations in substrate utilization in the myocardium after empagliflozin administration; however, we observed no effect on the utilization of ketone bodies in the myocardium. According to the authors of one study involving mice with heart failure, ketone bodies may contribute to the anti-inflammatory effect of empagliflozin in the heart (Byrne, et al., 2020). However, in our study, we found that the possible anti-inflammatory effects of empagliflozin related more to the generation of anti-inflammatory lipid metabolites and anti-inflammatory cytokines in cardiac tissue.

Moderate weight loss may also indirectly contribute to a reduction in cardiovascular impairment after empagliflozin treatment. In our study, empagliflozin reduced both total body weight and visceral adipose tissue weight. Interestingly, whole body weight and visceral adipose tissue weight loss occurred despite a slight increase in food intake after empagliflozin administration, a finding supported by other studies (Petito-da-Silva et al., 2019; Hojna, et al., 2021). Reduced visceral adipose tissue is probably the main mechanism involved in body weight loss. Nevertheless, other mechanisms such as increased basal metabolism (Kim, et al., 2018) and increased body temperature (Xu et al., 2017) may also contribute to weight loss following treatment with SGLT2 inhibitors. According to a recent study, empagliflozin may reduce epicardial adipose tissue mass (Sato et al., 2018), indirectly contributing to a reduction in myocardial inflammation. Our study excluded measurements of subcutaneous and epicardial adipose tissue mass. However, adipose tissue mass and metabolic activity may have an impact on cardiovascular pathology.

According to recent studies (Petito-da-Silva et al., 2019; Nasiri-Ansari et al., 2021) and our research (Huttl et al., 2021), empagliflozin can ameliorate NAFLD development, which is one of several independent cardiovascular risk factors. In one experimental model exhibiting fatty liver, empagliflozin treatment reduced hepatic lipogenesis and the accumulation of ectopic lipids and lipotoxic intermediates. In our study, although these effects were roughly comparable in the liver and in the heart, the effect of empagliflozin on lipid metabolism, reflected in the production of lipid metabolites, was more pronounced in the heart than in the liver. It may be the case that empagliflozin primarily influences lipogenesis in the liver, even though its effect on this organ was less potent than its effect on the heart in our study. In our previous study (Huttl et al., 2020), empagliflozin modulated the expression of genes involved in lipogenesis and lipid storage, including *Scd-1*, *Fas*, *Srebp1*, and *Pparγ*, in the liver. However, genes involved in lipid oxidation were not affected. To our knowledge, no study has compared the effects of empagliflozin on lipid metabolism in the heart and in the liver, and moreover, myocardial lipid metabolism was monitored in relation to myocardial ketone body metabolism. On the other hand, cardiac functions were not directly measured in our study, nor were changes in myocardial structure directly monitored. Furthermore, it is important to note that the alterations in lipid metabolism are part of the pleiotropic effects of SGLT2 inhibitors, and other mechanisms such as affecting hemodynamic parameters, including decrease in blood pressure and extracellular volume, have a significant cardioprotective effect associated with the SGLT2 inhibitors treatment (Abdul-Ghani et al., 2016).

## 5 Conclusion

In summary, empagliflozin may exert cardioprotective effects by positively influencing both myocardial and hepatic lipid metabolism and reducing inflammation in the heart. Empagliflozin decreased the accumulation of ectopic lipids and lipotoxic intermediates and reduced lipogenesis. We speculate that the anti-inflammatory and antifibrotic effects of empagliflozin are attributed to its influence on the metabolism of n-3 PUFA and arachidonic acid in the heart. The cardioprotective effects of empagliflozin might be connected with alterations in myocardial and hepatic lipid metabolism in prediabetic states with severe dyslipidemia.

## Data Availability

The original contributions presented in the study are included in the article/supplementary material, further inquiries can be directed to the corresponding author.

## Abbreviations

AA, arachidonic acid; BHB, β-hydroxybutyrate; BDH1, β-hydroxybutyrate dehydrogenase 1; hsCRP, high-sensitivity C-reactive protein; DAG, diacylglycerol; DHA, docosahexaenoic acid; D9D, delta-9 desaturase; EET, epoxyeicosatrienoic acid; EPA, eicosapentaenoic acid; FAS, fatty acid synthase; FA, fatty acid; FADS, fatty acid desaturase; HETE, hydroxyeicosatetraenoic acid; HHTg, hereditary hypertriglyceridemic; IL-6, interleukin 6; IL-10, interleukin 10; MCP-1, monocyte chemoattractant protein-1; NAFLD, nonalcoholic fatty liver disease; NEFA, nonesterified fatty acid; NFκB, nuclear factor kappa-B; PC, phosphatidylcholine; PE, phosphatidylethanolamine; Pparγ, peroxisome proliferator-activated receptor gamma; SCD-1, stearoyl-CoA desaturase 1; SREBP1, sterol regulatory element-binding protein 1; SGLT2, sodium–glucose cotransporter 2; TAG, triacylglycerol; TNFα, tumor necrosis factor alpha.
